# The burden of incontinence in a real-world data environment—insights from a digital patient companion

**DOI:** 10.1007/s00192-021-04683-4

**Published:** 2021-03-11

**Authors:** Alexandra von Au, Stephanie Wallwiener, Lina Maria Matthies, Benjamin Friedrich, Sabine Keim, Markus Wallwiener, Christl Reisenauer, Sarah Brugger

**Affiliations:** 1 Department of Obstetrics and Gynecology, University of Heidelberg, Heidelberg, Germany; 2 Temedica GmbH, Munich, Germany; 3 Department of Diagnostic and Interventional Neuroradiology, Technical University Munich, Munich, Germany; 4 Department of Obstetrics and Gynecology, Helios Klinikum München West, Munich, Germany; 5 Department of Obstetrics and Gynecology, University of Tuebingen, Tübingen, Germany; 6 Department of Obstetrics and Gynecology, Rotkreuzklinikum München, Munich, Germany

**Keywords:** Urinary incontinence, Pelvic floor training, Physiotherapy, App

## Abstract

**Introduction and hypothesis:**

Urinary incontinence (UI) has a potentially devastating effect on women’s quality of life (QoL). Conservative treatment by means of pelvic floor muscle training is the first-choice treatment modality. Nowadays, this can be supported by digital apps like pelvina©—a digital health companion pelvic floor course.

**Methods:**

Using pelvina©, UI symptoms and QoL are regularly examined through the questionnaires QUID and SF-6D. Subsequently, we analyzed the incidence and degree of UI and its impact on QoL in 293 users in a real-world environment.

**Results:**

The 293 patients included in this study had a median age of 36 years and a median of two children. Patients were slightly to moderately affected by UI with a QUID of 6 (2–11, maximum 24). Age and number of children were independently associated with the incidence of UI with an adjusted odds ratio (aOR) of 1.06 (95% CI 1.01–1.12) and aOR of 1.86 (95% CI 1.12–3.08). The severity of UI strongly correlated with impairment of QoL (ρ = 0.866, *P* < 0.001).

**Conclusions:**

The use of real-world data generated by digital health solutions offers the opportunity to gain insight into the reality of patients’ lives. In this article, we corroborate the known associations between number of children and UI as well as the great influence UI has on QoL. This study shows that, in the future, the use of digital apps can make an important contribution to scientific data acquisition and, for example, therapy monitoring.

## Introduction

Pelvic floor dysfunction (PFD) or pelvic floor disorders are general terms used to describe conditions that compromise the female continence mechanism (urinary and fecal) and/or pelvic organ support. It is common in women, especially with advancing age [1]. Pelvic floor integrity is maintained by the coordinated actions of muscles (e.g., levator ani and urethral and anal sphincters), nerves (sacral plexus and pudendal nerve), and connective tissue (e.g., endopelvic ‘fascia’, perineal body) anchored to the bony pelvis [2]. Pelvic floor muscle function can be qualitatively defined by muscle tone at rest and the strength of a voluntary or reflex contraction as strong, normal, weak, or absent [3]. The definition of PFD in women is usually limited to include only urinary incontinence (UI) and pelvic organ prolapse (POP). However, atrophic changes in the lower urinary tract can lead to restrictions in sexual function, dysuria (painful or difficult urination), and recurrent genitourinary tract infection as well [4]. UI can be divided into stress urinary incontinence (SUI) and urgency urinary incontinence (UUI). In SUI, physical exertion can be associated with urinary loss. Increased intra-abdominal pressure triggered by physical exertion increases intravesical pressure, and if it exceeds the intraurethral pressure in the absence of contraction of the detrusor muscle, the resulting urinary leakage is referred to as SUI. Thus, SUI is the complaint of involuntary urine loss on effort or physical exertion (also on sneezing or coughing) while UUI is the complaint of involuntary loss of urine associated with urgency [5]. Mixed forms of incontinence often occur and aggravate diagnosis and therapeutic concepts. According to different studies, UI has a devastating effect on women’s quality of life (QoL) in the physical, social, sexual, and psychological spheres [6]. Women restrict or diminish their activity and social participation, which has serious implications [7].

While UI is common in elderly women, also younger women can suffer from it. During the second and third trimesters of pregnancy and in the first 3 months following childbirth, about one-third of women experience UI [8]. During pregnancy and after delivery, the strength of the pelvic floor muscle (PFM) may decrease as a result of hormonal and anatomical changes (in both the position of the pelvis and the shape of the PFM), facilitating musculoskeletal alterations that can lead to UI. The overall prevalence (all types of UI) during pregnancy is estimated to be around 58%, and SUI affects about 31–42% of either childless women (nulliparous) or women with children (multiparous) [9]. In clinical practice, nonsurgical therapies are usually tried first because they are likely to carry the least risk of harm. The EAU Guidelines for Urinary Incontinence in Adults recommends supervised intensive pelvic floor muscle training (PFMT), lasting at least 3 months, as a first-line therapy for all women with SUI or mixed urinary incontinence (including the elderly and postpartum patients) [3]. However, as the topic of UI is embarrassing for most women even when talking to health care personnel, treatment options frequently are not carried out or are canceled prematurely by the patients [3].

Owing to technical developments in the last few years, patients are no longer exclusively dependent on treatment or instruction by physicians and physiotherapists, which, as mentioned above, on the one hand represents a high hurdle due to a possible feeling of shame and, on the other, is often difficult to organize in terms of time. Today, there is an increasing market for digital solutions that support patients for a wide range of indications, including back pain [10], psychiatric disorders [11], cancer [12, 13], and UI [14]. In addition, eHealth solutions also provide an insight into the daily lives of users and are suitable for the collection of real-world data (RWD). With the help of Temedica’s “pelvina©” app, which is one of these new tools, patients can train their pelvic floor in a comfortable and safe environment and thus become active on a regular basis both preventively and as an intervention. Giving patients this option to perform PFMT in a more personal and anonymous environment is of key importance to reduce treatment barriers for UI. pelvina© is the first German mobile app designed for iOS or Android devices and was certified by “Zentrale Prüfstelle Prävention” (ZPP) as a pelvic floor course for the prevention of pelvic floor-related complaints such as UI. As part of this app, users are given advanced knowledge about and targeted exercises against pelvic floor weakness. Furthermore, they are regularly asked about their UI status and the influence of UI on their QoL. Thus, for the first time, such new tools offer insight into real-world data beyond classical studies.

The primary aim of this study was to evaluate whether digitally collected patient-generated data from pelvina© can reproduce findings from previous studies concerning the impact of incontinence on QoL in a real-world data setting. As pelvina© is a tool also focusing on the prevention of pelvic floor weakness by providing information, a secondary aim was to analyze how well the patients had already been informed about pelvic floor weakness.

## Methods

**Fig. 1:**
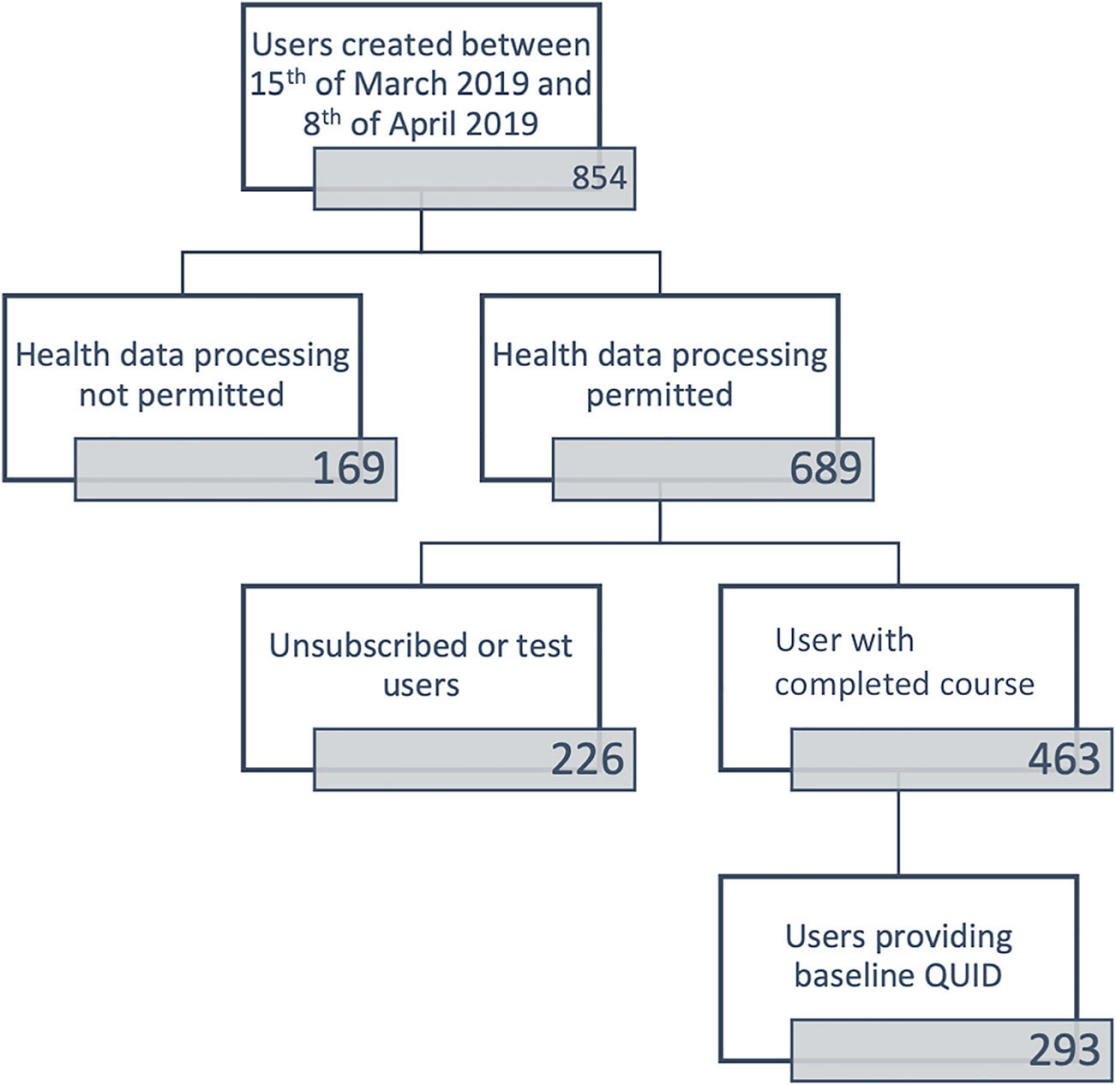
Consort chart of the excluded and included patients in the present study

For the present analysis, we included patients subscribing to pelvina© between March 15, 2019, and April 8, 2019, who gave consent to the onboarding process of the app to evaluate their data and for whom datasets were complete. From initially 854 users 169 did not give informed consent, 226 users unsubscribed before completing the course, and 170 users did not or incompletely answer the baseline QUID (see Fig. 1). Consequently, these 561 participants had to be excluded from our study. All remaining datasets were analyzed in a completely anonymized manner. The present study was approved by the ethics committee of the Heidelberg University Hospital (S-392/2019).

### Data acquisition and pelvina©

Inclusion criteria in the present study were:

- Usage of pelvina
- Minimum age of 18 years
- Consent to data evaluation during the app onboarding process
- Complete datasets

No further inclusion or exclusion criteria were applied.

According to § 20 SGB V in Germany, pelvina© (Temedica, Munich, Germany) is a certified pelvic floor course (certified by the ZPP) for the prevention of pelvic floor-related complaints such as UI. German health insurances pay up to 100% of the costs. It is available via smartphone app (iOS and Android). When starting the app the onboarding process begins. Here demographic data are collected via a chatbot dialog (see Table 1). The participants' medical history is also queried to allow a more targeted training. In eight thematic blocks, the participants learn how the pelvic floor is structured and how it works. Furthermore, they are given exercises to help become sensitized to it, strengthen it, and protect it in everyday life. The training program takes place anonymously at the participants’ homes, including instruction by the course instructor via video. After each module, the user receives a summary of the educational content as well as a training manual as a PDF document by e-mail. As soon as a module has been completed, a new module is activated, and the user can set the pace herself.

**Table 1:** Data collection

Basic visit	Visits 2–8
Beginning of the course, week 0	
Demographic data	
Medical history	
Questionnaires • QUID • QUID	Questionnaires • SF-6D • SF-6D

Furthermore, in each block of the course the participants are asked to fill out the Questionnaire for Urinary Incontinence Diagnosis (QUID), a six-item UI symptom questionnaire that was developed and validated to distinguish and evaluate stress and urgency UI [15]. Three items focus on stress incontinence symptoms and three on urge incontinence symptoms. Each item includes six frequency-based response options, ranging from “none of the time” to “all of the time,” which are scored from 0 to 5 points. Scores are calculated in an additive fashion, resulting in separate stress and urge scores, each ranging from 0 to 15 points. Therefore, a grading between 0 and 30 scale points is possible, with larger numbers indicating a greater severity of incontinence symptoms.

Additionally, the QoL of the patients was analyzed in every block of the course using the SF-6D, a well-established six-item QoL outcome measure [16]. Results from 0 to 30 are possible, with larger numbers indicating a larger impairment of QoL.

For the present study, the dataset was extracted from the pelvina© database. Data anonymization is implicit by not including any personal data in the export, so it is impossible to retrospectively identify a user based on their records in the export. Gradually increasing numbers are used to identify users in the export. This anonymized dataset was then analyzed.

### Statistical analysis

Statistical analysis was performed using SPSS 25 (IBM, Armonk, NY, USA). Correlation between the various parameters was assessed using Spearman’s ρ correlation. The difference between the rate of previous knowledge about pelvic floor insufficiency and the number of births was analyzed using ANOVA for ranking followed by a Tukey post-hoc analysis with bootstrapping with 10,000 permutations. After univariate analysis by a Mann-Whitney U test, a multivariate logistic regression analysis was performed to test the association between age and number of children on the incidence of incontinence. All data are presented as median (interquartile range) if not indicated differently. Results derived from the logistic regression analysis are shown as the adjusted odds ratios (aOR) and respective 95% confidence intervals (95% CI). Statistical significance was assumed at *P* < 0.05.

## Results

### Patient characteristics

A total of 293 patients fulfilled all the previously defined inclusion criteria (Fig. 1). The median age was 36 years, and the patients had given birth to two children on the median. Sixteen percent of the patients (*n* = 47 of 293 patients) reported comorbidities, e.g., a history of cancer or diabetes mellitus, and 25% of the patients (*n* = 73 of 293 patients) reported regular medication usage. In those cases, hormone therapy was most often used, with 12% of the patients (*n* = 35 of 293 patients) taking some form of regular hormone therapy (either contraceptive or hormone replacement therapy). Furthermore, 4.4% of the patients indicated that they were taking antihypertensive medication and 2.7% of the patients that they were taking regular medication for asthma. We were therefore able to indirectly conclude that the corresponding patients suffered from arterial hypertension or asthma (see Table 2).

**Table 2:** Patient characteristics

*n*	293
Median age (IQR)	36 (32–44)
Number of children	2 (1–2)
Comorbidities	
Cervical cancer	1.7%
Renal cancer	0.7%
Vaginal cancer	0.3%
Diabetes	1.4%
Multiple sclerosis	1%
Arterial hypertension	4.4%
Asthma	2.7%
Depression	1%
Other	10.6%
Regular medication	
Antihistamines	5.1%
Antidepressants	1%
Antihypertensive drugs	4.4%
Asthma drugs	2.7%
Hormone therapy (either anticontraceptive or HRT)	11.9%

*IQR* interquartile range, *HRT* hormone replacement therapy

### QUID and SF-6D

**Fig. 2:**
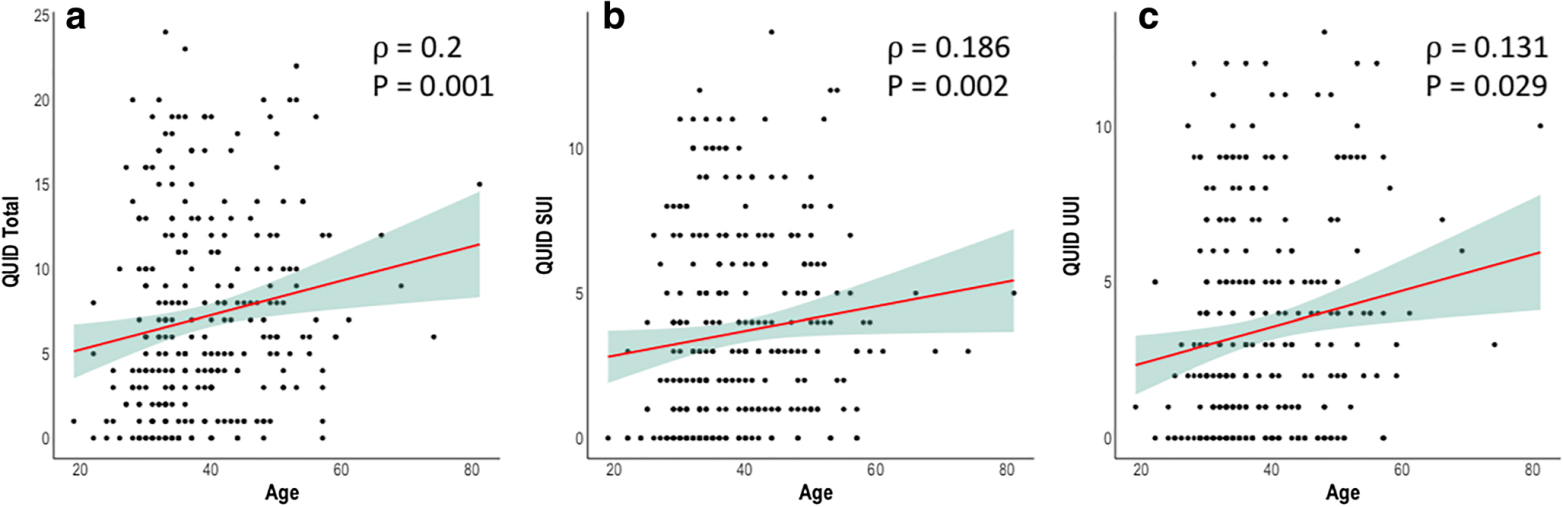
Correlation between patient age and urinary incontinency by means of the QUID (**a**) with the subdomains stress urinary incontinence (**b**) and urgency urinary incontinence (**c**)

**Fig. 3:**
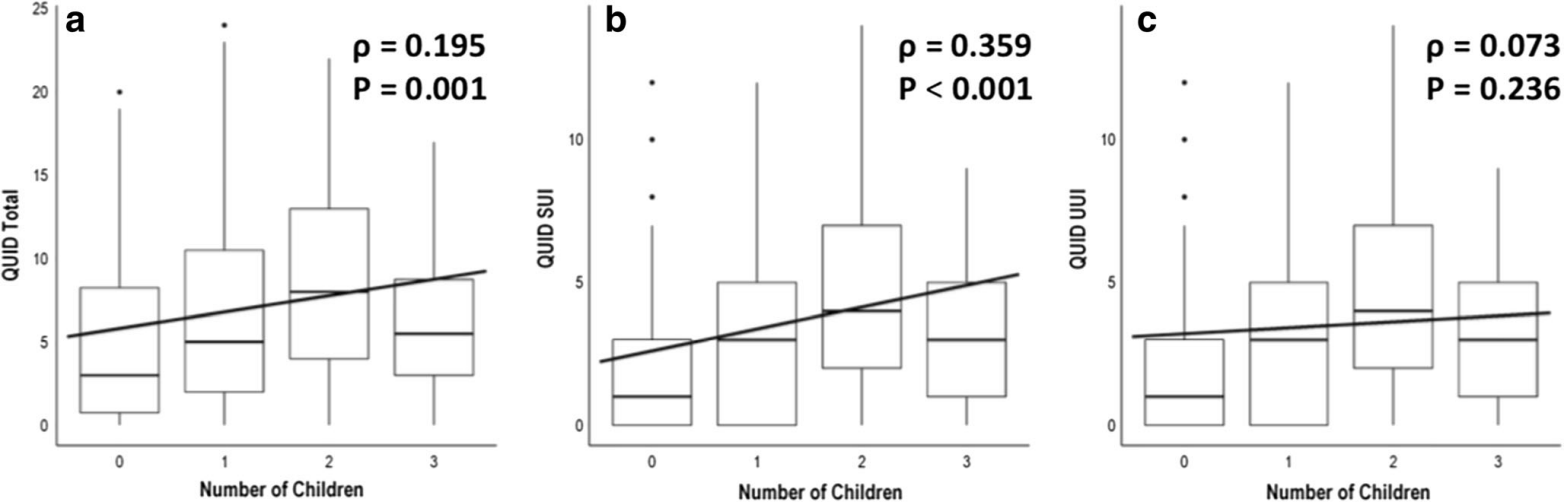
Correlation between number of children in our patient cohort and urinary incontinency by means of the QUID (**a**) with the subdomains stress urinary incontinence (**b**) and urgency urinary incontinence (**c**)

**Fig. 4:**
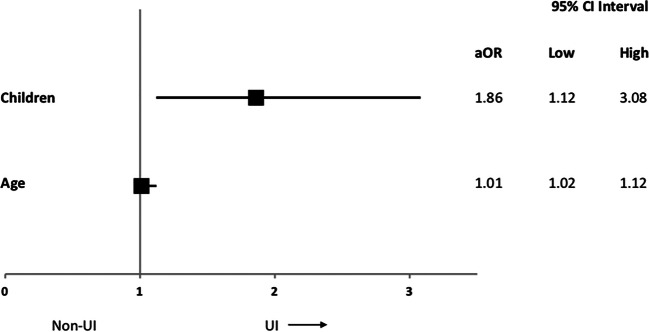
Forest plot showing the results of the logistic regression analysis

Regarding their incontinence, the patients reported slight to moderate incontinence symptoms in their QUID scale. The patients had a median score of 6 (2–11, maximum 24) at the beginning of the course at pelvina©. The patients were slightly more affected by stress incontinence symptoms with a median QUID_stress_ of 3 (1–6, maximum 14). In contrast, there were slightly less pronounced urgency incontinence symptoms with a median QUID_urge_ of 2 (0–6, maximum 13). We detected a positive correlation between the age of our patients and the severity of incontinence expressed by the QUID score (ρ = 0.2, *P* = 0.001) (Fig. 2). Additionally, we found a highly significant correlation between the number of children of the woman and the severity of incontinence symptoms expressed by the QUID score (ρ = 0.195, *P* = 0.001) (Fig. 3). This correlation could also be shown when regarding SUI and UUI separately (Fig. 3b and c). To analyze the interaction between number of children and age, we performed a multivariate logistic regression analysis for the incidence of incontinence with both age and number of children as covariates. Age predicted the incidence of incontinence with an adjusted odds ratio of 1.06 (95% CI 1.01–1.12), and the number of children was associated with the incidence of incontinence with an adjusted odds ratio of 1.86 (95% CI 1.12–3.08) (Fig. 4).

**Fig. 5:**
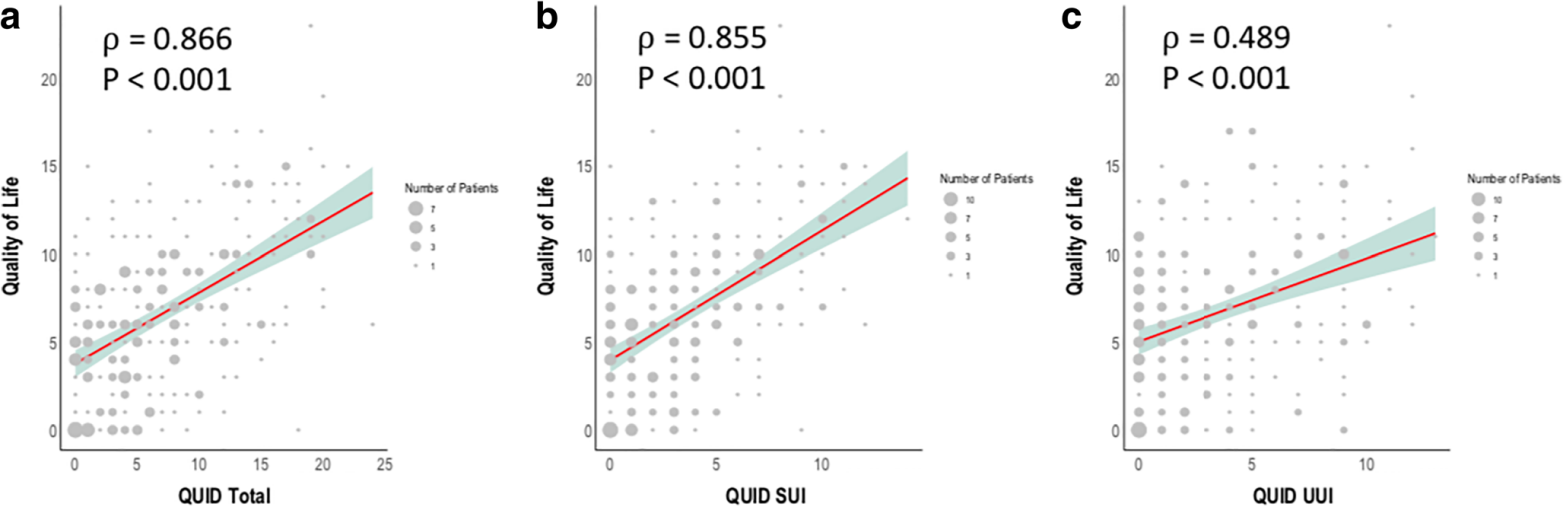
Correlation between quality of life (where higher numbers indicate a larger impairment of quality of life) and urinary incontinence by the QUID (**a**) with the subdomains stress urinary incontinence (**b**) and urgency urinary incontinence (**c**)

**Fig. 6:**
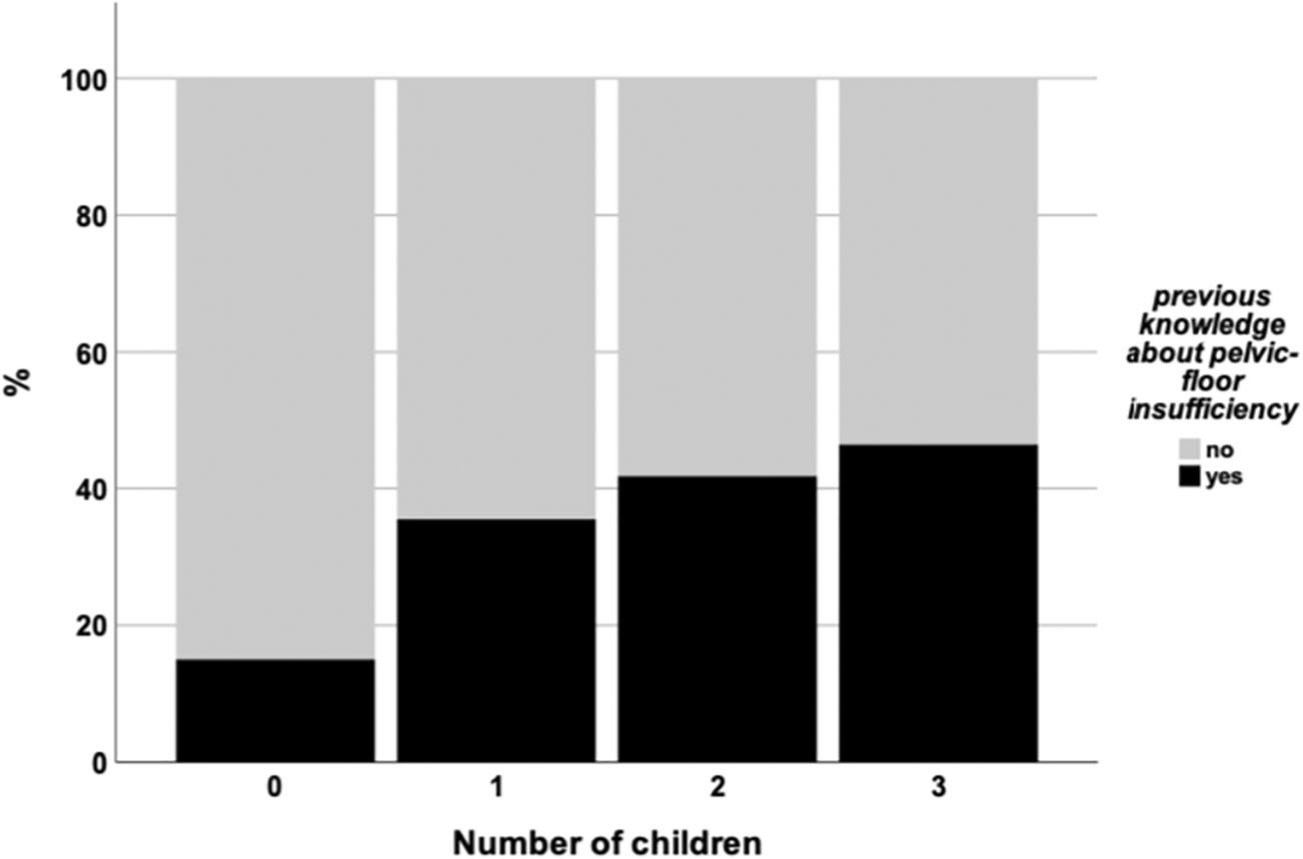
Rate of previous knowledge among patients using the presented app according to the number of children

Furthermore, we analyzed the impact of incontinence on QoL in a real-world setting using the SF-6D. There was a significant positive correlation between the severity of incontinence symptoms expressed by the QUID score and restrictions in QoL (ρ = 0.866, *P* < 0.001) (Fig. 5). This association was also true for symptoms from both the area of stress incontinence and urgency incontinence (QUID_stress_: ρ = 0.509, *P* < 0.001; QUID_urge_: ρ = 0.333, *P* < 0.001). Furthermore, there was a clear association between number of children and the rate of prior knowledge regarding the pelvic floor. While nulliparous women only had previous knowledge about the pelvic floor in 25% of the cases, general knowledge about the role of the pelvic floor rose to 46.4% after the third child (*P* = 0.014, Fig. 6).

## Discussion

In this retrospective observational study, we analyzed the rate of incontinence and its influence on the QoL in women using pelvina©—a digital health companion for pelvic floor disorders. There was a significant correlation between the age of the patients and incontinence on the one hand and the number of children and incontinence on the other. These findings are in line with the current literature. In one of the largest epidemiological surveys on urinary incontinence Hannestad and collegues—analyzing data from 27,936 women—found that the prevalence of urinary incontinence increased with increasing age [17]. Furthermore, a correlation between the number of children and incontinence has also been described before [18]. As pelvina© is a tool focusing on the prevention of pelvic floor weakness (e.g., underactive pelvic floor muscles) by training and providing information and as the number of deliveries—especially the number of vaginal deliveries—represents a major risk for acquired pelvic floor weakness, the question arose as to how well the patients had already been informed about the dysfunction of pelvic floor weakness. In our study cohort, the knowledge significantly increased with the number of children. This was not unexpected as in Germany patients are routinely recommended to participate in birth preparation courses where the patients are also provided information on the pelvic floor muscles. Moreover, we were able to show that, although in our cohort most of the patients only had mild to moderate incontinence, this had a strong impact on the QoL of affected patients, a result which was also reported by Hannestad et al. In the EPINCONT Study, 80% of the incontinent women suffered from their condition [17]. Additionally our patients with pelvic floor weakness showed more frequent symptoms of SUI than UUI, which is also in line with other studies [19].

Thus, the analysis of digitally collected real-world data confirms the published literature on incontinence and its influence on the QoL.

Traditional studies—whether observational studies or other study designs—have defined modern medicine and paved the way for evidence-based development. However, these classical studies are not without problems and difficulties as they suffer from restrictions.

The recruitment of patients is often extremely difficult and time-consuming in clinical practice. For this reason, it often takes a long time until studies have recruited a sufficient number of cases to be able to make valid statements. Due to this high expenditure of time and personnel, horrendous costs are often incurred—sometimes up to 6000 USD per included patient [20]. Furthermore, collecting data also involves considerable effort, be it personal interaction, telephone interviews, or written questionnaires by post. In particular, the sending of questions by post is associated with a relatively low response rate, for example, rarely more than 60% for the topic of UI [21, 22], which might bias the collected data. Not least for this reason, it is often difficult to collect longitudinal data within the framework of a classical study design over a longer period of time, being even more difficult for repeated data collection at frequent times over a long period. Thus, data collection in classical studies is often more of a snapshot at a certain time than a statement about a general condition. Many of these obstacles of classical studies can be overcome by using modern digital solutions. Indeed, by using digital solutions such as those available in app stores, a potentially global patient population can be reached. It is not necessary to interact directly with potential study participants and to recruit them from one’s own pool of patients. Thus, valid case numbers can be recruited in a very short period of time. In the present study, pelvina© required only 3 weeks of recruitment time, whereas other studies on QoL and UI often require 1 or more years to recruit a similar number of patients [23, 24]. In the field of UI in fertile patients, new circumstances such as further pregnancies that occur during the otherwise long process of data collection might interfere with the interpretation of the data. Another advantage of digital solutions lies in the fact that the collection of data is not necessarily linked to the personal effort of the investigators: patients and users can collect these data independently, for example, at home. Since this element frees up enormous resources on the part of the study management, completely new study designs are conceivable, such as very longitudinal surveys or very frequent data collections. From this perspective, such new means of acquiring data can give extremely relevant insights and findings and thus provide a better understanding of the disease under investigation. One other point that should not be neglected is that for the aforementioned reasons and the inherent, completely digital workflow (data collection, data transfer, data analysis), substantially lower costs are incurred when conducting a study in this manner.

In the present study, we were able to gain substantial insights into the rate of UI and its influence on QoL in an extremely short period of time using real-world data acquired by the pelvina© app. In analogy to published studies, we were able to prove that UI due to pelvic floor dysfunction has a clear association with the number of births in the real world, too, and that this is independent of the age of the patients. This is especially important in view of the fact that age is a known important predictor for the occurrence of UI [19]. We were also able to reproduce this finding using the digitally collected real-world data.

The limitations of the present study are the currently still relatively small number of patients, although highly significant results can still be derived here. However, due to the increasing number of users of the app used in this study, a rapidly growing number of cases is expected for future investigations. Furthermore, the present study design does not allow any conclusions to be drawn about the incidence of UI since, on the one hand, there is no control group and, on the other, an inherent bias is the fact that only patients who subjectively or objectively, according to medical consultation, suffer from pelvic floor dysfunction use pelvina©. Furthermore, the users of pelvina© are only asked about the number of children, not the number of deliveries. The delivery mode is also unknown. Therefore, it is impossible for us to correct for those potential confounders. In addition, of course, a retrospective analysis itself has many known inherent limitations. As is unfortunately typical for this study design, we had to exclude a relevant number of patients from the analysis because the datasets were incomplete. Furthermore, when using digital health companions for scientific investigations the examiners primarily have to rely on the information provided by the participants without the possibility of completing the information using the patients’ medical files or directly talking to the patients. As a result, we had to deduce some of the previous illnesses from the medication listed in the app.

## Conclusion

Our results confirm the massive influence of UI on the QoL of affected patients. Intensive physiotherapy and regular postpartum training are therefore clearly indicated to alleviate and treat complaints caused by pelvic floor weakness. A great advantage of digital solutions such as pelvina© is that continuous data acquisition makes it possible to quickly evaluate the benefits and effects of such therapeutic approaches. Further investigations need to be performed to examine whether the use of such digital tools can improve UI and the resulting influence on QoL.
